# Editorial: Responses of Tea Plants to Climate Change: From Molecules to Ecosystems

**DOI:** 10.3389/fpls.2020.594317

**Published:** 2020-11-24

**Authors:** Wenyan Han, Selena Ahmed, Chaoling Wei, Colin M. Orians, Marco Landi

**Affiliations:** ^1^Tea Research Institute, Chinese Academy of Agricultural Sciences, Hangzhou, China; ^2^Sustainable Food Systems Program, Department of Health and Human Development, Montana State University, Bozeman, MT, United States; ^3^State Key Laboratory of Tea Plant Biology and Utilization, Anhui Agricultural University, Hefei, China; ^4^Department of Biology, Tufts University, Medford, MA, United States; ^5^Department of Agriculture, Food and Environment, University of Pisa, Pisa, Italy

**Keywords:** *Camellia sinensis*, climate change, environmental stressors, plant defense, crop quality, abiotic stress, biotic stress, secondary metabolites

We find ourselves at a time when climate change is impacting almost every facet of society, including the sustainability of agricultural and food systems. The science is clear that climate change is happening and its impact will worsen without efforts to reverse the trend with more sustainable and eco-friendly approaches (IPCC, 2014). As scholars, practitioners, and citizens of this planet, we must collectively act to understand the risks posed by climate change and then to apply this knowledge for climate mitigation and adaptation. Science plays a key role in understanding climate effects on earth's systems toward co-designing evidence-based solutions. This Research Topic seeks to advance our understanding of the effects of climate change on the multiple dimensions of crops through the lens of the tea plant.

The tea plant, *Camellia sinensis* (L.) Kuntze is an ideal model perennial plant for examining climate effects on crops as it is the most widely consumed drink globally after water with notable cultural, dietary, and health values. Tea is cultivated on five continents in diverse types of agricultural systems and supports livelihoods and contributes to regional economies (Han et al., 2018). The consumption of tea is linked to its flavor and health attributes which are based on distinct secondary metabolite biochemical profiles comprised of polyphenols, amino acids, alkaloids, terpenoids, and other volatile compounds. Tea secondary metabolites are key determinants of quality that are sensitive to climate variability; this variability has notable implications throughout the tea system from farmer management to consumer preferences and tea sector livelihoods (Ahmed et al., 2014).

Articles in this Research Topic provide evidence on the effects of climate change on tea plants based on physiological, molecular, and biochemical responses, and ecosystem interactions and industry more broadly. Some articles in this Research Topic provide a survey of literature on existing evidence on the effects of global change on tea plants, whilst others propose new methodologies for understanding their impact or unveil novel findings on the topic. Collectively, the articles in this collection advance our understanding of the effects of various environmental stressors linked to climate change including water stress, salt stress, temperature fluctuations, solar radiation, herbivory, and pathogens. The evidence compiled in this collection demonstrates that tea plants react with different defense mechanisms including physiological, molecular, and biochemical responses to environmental stressors linked to climate change.

A unique emphasis of this Research Topic is the focus on the effects of climate on tea quality as determined by secondary metabolite biochemical profiles. While extensive research has focused on the effects of multiple stresses on crop yields, this collection also advances our understanding of their impact on crop quality. Below we provide a general overview of the collection of articles in this Research Topic, starting with the literature reviews and then the primary research articles. For the primary research articles, we first present articles focused on plant performance, and then those focused on the biochemical responses that influence tea quality.

The three literature reviews in this Research Topic highlight what we know about climate effects on tea and elucidate current gaps in research. With an emphasis on the plant performance aspects of tea production, Muoki et al.'s review compiles evidence on the varied responses of tea plants to climate change with a focus on the Kenyan tea industry. They synthesize strategies for conventional and molecular breeding and selection toward informing climate resilient tea cultivation. With a focus on the biochemical responses of tea plants, Ahmed et al.'s systematic review synthesizes the effects of environmental factors on tea quality from 86 articles, carried out in tea producing regions globally. This systematic review demonstrates that shifts in water stress, seasonality, geography, light factors, altitude, herbivory and microbes, temperature, and soil factors can result in both increases and decreases of up to 50% in tea secondary metabolites. A key gap in the literature elucidated through this review is a lack of studies examining the effects of elevated CO_2_ on tea quality. Ahammed et al. build on this finding through a review characterizing the physiological and metabolic responses of tea plants to elevated CO_2_ including alterations in concentrations of polyphenols, free amino acids, catechins, theanine, and caffeine as well as associated gene expression.

Drought, low temperatures, pests and diseases, salt, and light are key factors that determine plant performance in existing and new areas of production (Han et al., 2018). At the morpho-anatomical level, the plant cuticle plays a major role in regulating water loss and drought tolerance, an attribute that is essential for climate adaptation in many tea-producing areas. Zhang Y. et al. propose a novel method that can be applied for measuring water loss through the leaf cuticle toward understanding drought tolerance and selecting drought tolerant tea varieties. The implementation of this method can potentially be applied to other plants to examine cuticular transpiration in the context of climate-change-promoted drought events.

Cold stress is a key environmental factor that adversely impacts plant growth and is a major factor limiting the expansion of tea cultivation (Chinnusamy et al., 2007). Low temperatures can result in the accumulation of reactive oxygen species in tea plants which in turn can promote the antioxidant capacity including the synthesis of flavonoids (Li et al., 2018). Glycosyltransferases (UGTs) are involved in the transport of flavonoids and render them more water soluble and less toxic (Song et al., 2018). Zhao et al. identify a UGT gene involved in the regulation of plant cold stress tolerance.

At the molecular level, microRNAs (miRNAs) are recognized as key modulators of gene expression in response to various environmental stressors (Khraiwesh et al., 2012). Jeyaraj et al. provide evidence on the role of miRNAs in tea plants in response to the plant pathogen *Colletotrichum gloeosporioides*. Findings from this study have implications for understanding pathogen susceptibility and resistance in tea plants. Wan et al. highlight how salt stress severely affects the growth and quality of tea and examine the molecular mechanisms involved in responses to salt stress. Specifically, they provide evidence on the roles of long non-coding RNAs (lncRNAs) in transcriptional regulation as ubiquitous regulators in response to salt stress in tea plants. Liu et al. examine how light is a key environmental regulator of the chlorophyll biosynthesis pathway in higher plants and provide evidence on the molecular mechanisms that regulate chlorophyll biosynthesis in response to light and hormones. At the ecosystem level, Zhang Q. et al. examine cultivars that are suitable for adaptation to a new tea producing areas in the United States by examining cold tolerance along with plant growth, morphology, and biochemical profiles. Together, these studies provide a model for selecting and developing crop cultivars that can be adaptable to new agricultural areas or changing conditions.

The biochemical plant responses linked to secondary metabolite synthesis are important to both plant resistance and plant quality. Five articles in this Research Topic focus on the shifts in various classes of secondary metabolites that influence tea flavor in response to multiple environmental stressors. Shamala et al. demonstrate how ultraviolet-B (UV-B) radiation stimulates the biosynthesis of phenolic compounds. Huang et al. shed insight on the biosynthesis of gallic acid, which is a precursor for polyphenol synthesis in tea plants. They provide experimental evidence from enzyme assays and kinetic analysis on favorable candidate genes for gallic acid biosynthesis in tea plants and connections of gallic acid biosynthesis to the shikimate pathway. Li et al. present a study examining the interplay of the secondary metabolite theanine, which contributes an umami taste to tea, and gene expression in diverse tea cultivars in the context of seasonal variability. They provide evidence that theanine fluctuations are both season- and developmental stage-dependent. Furthermore, they provide evidence that while theanine-biosynthesis genes are generally negatively correlated with theanine content, amino acid transporter genes are generally positively correlated with theanine accumulation.

Kfoury et al. provide evidence on the variation of secondary metabolites based on spatial and temporal factors. They demonstrate that elevation is a key determinant of crop quality based on the profiling of over 500 volatile secondary metabolites in one tea producing area of China, Yunnan Province, but not in another, Fujian Province. They show that seasonality and the annual variation associated with changing environmental conditions are determinants of crop quality in both locations. Scott et al. provide evidence on the relationship of increasing leafhopper density in tea agricultural systems to damaged leaf area and both volatile and non-volatile secondary metabolites through a manipulative experiment. Both Kfoury et al. and Scott et al. show that induced biochemical responses to environmental factors can be linear or non-linear. For example, Scott et al. show that some secondary metabolites change linearly with herbivore pressure, and that others respond after reaching a specific threshold following herbivore pressure.

Collectively, the findings of the articles in this Research Topic have notable implications for the resilience of crops and agricultural systems in the context of global change. While the articles in this Research Topic focus on tea, we expect that they will provide methodologies and insights on examining the responses of other agriculturally- and economically-important plants to climate change. This Research Topic further highlights the critical role of plant sciences in understanding responses to global change and designing sustainable and resilient crop systems. To reduce the detrimental impact of climate change is not an easy task, however, if we do not take rapid actions, the world will be less habitable. Cooperation between researchers, practitioners, producers, and policy stakeholders is essential to co-design and implement evidence-based mitigation and adaptation strategies for tea and, more generally, for plant cultivation.

## Author Contributions

SA and WH wrote the draft of the editorial, with all co-authors jointly editing the final version.

## Conflict of Interest

The authors declare that the research was conducted in the absence of any commercial or financial relationships that could be construed as a potential conflict of interest.
